# Salicylic Acid Is Involved in the Basal Resistance of Tomato Plants to Citrus Exocortis Viroid and Tomato Spotted Wilt Virus

**DOI:** 10.1371/journal.pone.0166938

**Published:** 2016-11-28

**Authors:** M. Pilar López-Gresa, Purificación Lisón, Lynne Yenush, Vicente Conejero, Ismael Rodrigo, José María Bellés

**Affiliations:** Instituto de Biología Molecular y Celular de Plantas, Universitat Politècnica de València (UPV)- Consejo Superior de Investigaciones Científicas (CSIC), Valencia, Spain; Gyeongnam National University of Science and Technology, REPUBLIC OF KOREA

## Abstract

Tomato plants expressing the *NahG* transgene, which prevents accumulation of endogenous salicylic acid (SA), were used to study the importance of the SA signalling pathway in basal defence against Citrus Exocortis Viroid (CEVd) or Tomato Spotted Wilt Virus (TSWV). The lack of SA accumulation in the CEVd- or TSWV-infected NahG tomato plants led to an early and dramatic disease phenotype, as compared to that observed in the corresponding parental Money Maker. Addition of acibenzolar-S-methyl, a benzothiadiazole (BTH), which activates the systemic acquired resistance pathway downstream of SA signalling, improves resistance of NahG tomato plants to CEVd and TSWV. CEVd and TSWV inoculation induced the accumulation of the hydroxycinnamic amides *p*-coumaroyltyramine, feruloyltyramine, caffeoylputrescine, and feruloylputrescine, and the defence related proteins PR1 and P23 in NahG plants earlier and with more intensity than in Money Maker plants, indicating that SA is not essential for the induction of these plant defence metabolites and proteins. In addition, NahG plants produced very high levels of ethylene upon CEVd or TSWV infection when compared with infected Money Maker plants, indicating that the absence of SA produced additional effects on other metabolic pathways. This is the first report to show that SA is an important component of basal resistance of tomato plants to both CEVd and TSWV, indicating that SA-dependent defence mechanisms play a key role in limiting the severity of symptoms in CEVd- and TSWV-infected NahG tomato plants.

## Introduction

The inability of higher plants to escape from a broad range of inevitable exogenous biotic and abiotic challenges has led to the development of very sophisticated defence mechanisms to efficiently survive a large variety of potential aggressions and hostile environments [1, 2]. Because of these elaborate defence systems, in the case of biotic stresses, only successfully evolved microorganisms can cause disease. These defence strategies consist of passive and preformed physical and chemical barriers and also of a system of active defence reactions that are elicited by phytopathogens. Two categories of plant-pathogen interactions can be broadly defined: compatible and incompatible. The encounter between the plant and a pathogen in an incompatible gene-for-gene type of interaction leads to the rapid collapse and cell death in and around the entry point of the pathogen, and the pathogen is confined to the infection site. In a compatible, non-necrotizing interaction, characterized by the absence of gene-for-gene resistance, the susceptible plant presents a much weaker and slower response. This type of interaction typically leads to the onset of disease symptoms in the inoculated tissue, which may progress throughout the entire plant. Although differing in the temporal expression pattern, some common resistance responses in both compatible and incompatible interactions include the synthesis of pathogenesis-related proteins, signal molecules such as salicylic acid (SA, 2-hydroxybenzoic acid) and natural antimicrobial products from the phenylpropanoid pathway including hydroxycinnamic acid amides (HCAA).

The plant hormone SA, a natural simple phenolic compound which is broadly distributed in higher plants, is a fundamental component of the signal transduction pathway that triggers defence responses against different invading pathogens in many species [3–5]. SA can be further hydroxylated to form gentisic acid (GA, 2,5-dihydroxybenzoic acid), which accumulates to high levels mainly in compatible interactions [6]. GA induces specific defence proteins that are not induced by SA suggesting that, in addition to SA, GA could act as a signalling molecule in plant defence responses [7].

The accumulation of HCAA in some plant-pathogen interactions indicates the importance of these compounds in plant defence. Specifically, challenging Rutgers tomato with *P*.*syringae* pv.*tomato*, rapidly increase the levels of hydroxycinnamoyl amides of tyramine, dopamine, octopamine and noradrenaline, which show effective antioxidant properties [8, 9].

Tomato (*Solanum lycopersicum* L.) has been extensively used as model host for studying physiological and biochemical aspects of compatible interactions, including those induced by the causal agent of the exocortis disease of citrus [9–13]. It has been found that tomato plants infected by the Citrus Exocortis Viroid (CEVd) accumulate high levels of both salicylic (SA) and gentisic acid (GA). Both phenolics might act as signals for the activation of the tomato plant defence response to different pathogens [14–17].

The viral pathogen Tomato Spotted Wilt Virus (TSWV), causes serious disease worldwide in tomato and other agronomic herbaceous plants [10]. Tomato plants inoculated with TSWV also results in the establishment of a compatible interaction, however the accumulation of phenolic compounds in this system has not yet been fully explored [11, 12].

NahG tomato plants express a salicylate hydroxylase gene from *Pseudomonas putida* that metabolizes SA to catechol and, consequently, these plants are unable to accumulate SA [13, 14]. These transgenic plants are excellent tools to study the key role of SA in the complex resistance response of the plant [15–18]. For example, this model system has been used to show that SA is an important component of the signalling pathway in the interactions of tomato plants with aphids [19] or nematodes [20, 21]. However, the role of SA in susceptible responses against virulent pathogens has not been extensively studied [22–24], and is still unexplored in plant-viroid interactions. Another convenient tool used to study the role of SA in plant defence responses is the physiologically active SA analogue benzothiadiazole (BTH). This compound is a synthetic, non-phytotoxic plant defence activator and a commercially-attractive disease control candidate [17, 25–27] used in field studies to combat bacterial diseases of tomato [28–30].

In this work, NahG tomato plants and BTH treatments have been used to investigate whether SA plays a biological role in the basal resistance of tomato plants against the subviral and viral tomato pathogens CEVd and TSWV.

## Materials and Methods

### Plant material and inoculation procedures

Transgenic tomato (*Solanum lycopersicum*) plants overexpressing the bacterial salicylate hydroxylase transgene (*NahG* gene) and the cultivar Money Maker, the isogenic parental line of NahG, were included in all experiments. Seeds from Money Maker and NahG plants were kindly provided by Professor J.D.G. Jones (John Innes Centre, Norwich, U.K.), and the procedure used to obtain NahG plants was previously described [13, 14]. The seeds were surface-sterilized with sodium hypochlorite and experimental lots with appropriate numbers of plants from each cultivar of uniform morphological and physiological conditions were prepared for each experiment. Money Maker and NahG tomato plants were grown in 20-cm-diameter pots (one per pot) containing a mixture of peat and vermiculite (1:1, w/w). The pots were subirrigated periodically when needed with a nutrient Hoagland solution.

For CEVd experiments, a total of fifty Money Maker and fifty NahG tomato plants were grown in a greenhouse with ambient lighting (300 μE/m^2^sec^-1^) supplemented by metal halide (Osram Powerstar 400 W HQI-BT/D Daylight E40) and sodium vapor (Philips MASTER SON-T PIA 400 W E40) lamps during a 16 h photoperiod, and a regime of temperature of 30/26°C (day/night), with a relative humidity ranging from 60% (day) to 85% (night). Half of the plants were mock-inoculated and the rest were infected with CEVd (GenBank accession number S67446), by inoculating both cotyledons of 2 week-old plants. The inoculum was prepared from CEVd-infected Rutgers tomato plants using leaves with severe symptoms as previously described [31]. Cotyledons were abraded with carborundum (particle size 0.037 mm), and inoculated by applying 50 ng of CEVd extract to the cotyledons and gently rubbing the adaxial surface. Cotyledons of control plants were abraded and mock-inoculated with 50 μl sterile demineralized water. After inoculation, plants were maintained in the greenhouse conditions as described above. The tip tissues consisting of apex and the new young leaves from mock and CEV-inoculated plants, showing mild to severe symptoms of epinasty and rugosity of the leaves and dwarfing, were sampled for all the analytical measurements at different time points during the infection.

Forty Money Maker and forty NahG tomato plants used for experiments with TSWV were grown in a controlled plant growth chamber (light intensity of approximately 70 μE/m^2^sec^-1^) with day and night temperatures of 24°C and 18°C, respectively, a day/light cycle of 16/8 hours and a relative humidity ranging from 70% (day) to 80% (night). Under this light regime, NahG plants do not exhibit a necrotic phenotype and TSWV actively replicates. Half of the plants were mock-inoculated and the rest were infected with TSWV according to Soler et al. [32]. Briefly, one gram of TSWV-infected leaves showing strong symptoms was homogenized in 20 ml of phosphate buffer (3 mM sodium monophosphate, 75 mM sodium diphosphate) (pH 7.4) supplemented with 0.15 M NaCl, 1% polyvinylpolypyrrolidone, 0.02% mercaptoethanol, 1% carborundum, and 1% active carbon. Four-week-old Money Maker and NahG plants were inoculated on the uppermost of all the leaflets from the third and fourth leaves (leaf position numbered from the base to the apex) with TSWV (50–100 μL of viral extract per leaflet). Plants were dusted with carborundum (particle size 0.037 mm) to aid pathogen entry into the leaf cells, and then inoculated by rubbing with a cotton swab soaked in inoculum or mock-inoculated with buffer alone. One week later, the fifth and sixth leaves were also inoculated as described above with the virus or with buffer. The fifth and sixth leaves were sampled for all the indicated analytical measurements at the different time points of the infection. Money Maker and NahG plants were inspected visually for evaluation of symptoms and the disease severity was scored 2, 2.5, 3 and 4 weeks post-inoculation in CEVd-infected plants, and 0.5, 1 and 2 weeks after inoculation with TSWV using the following scale: symptomless, moderate, severe, and very severe.

### Treatment of Money Maker and NahG tomato plants with BTH

The BTH derivative used in this work (acibenzolar-S-methyl) was provided as the trademark Bion 50 WG, 50% w/w active ingredient concentration, by Syngenta Crop Protection AG, Basel, Switzerland. BTH solution was prepared immediately before spraying at a concentration of 1 mM supplemented with 0.05% (v/v) Tween 20. The suspension was applied uniformly as a fine mist with a hand-held sprayer until the suspension ran off the leaf surfaces. To study its protective effect against CEVd infection, half of the Money Maker and NahG tomato plants infected with CEVd were sprayed with the BTH solution at days 3 and 6 after inoculation. The effect of BTH on TSWV-infected plants was investigated by treating Money Maker and NahG plants with BTH at day 3 after the first inoculation and again at day 3 after the second inoculation with the virus. Equivalent control mock-inoculated Money Maker and NahG tomato plants were treated at the same time points with 0.05% (v/v) Tween 20 in demineralized water only.

### Extraction and HPLC analysis of SA and GA

Levels of free and total SA and GA were monitored in extracts from the appropriate leaf tissues of CEVd- and TSWV-infected Money Maker or NahG tomato plants and the corresponding mock-inoculated controls at different times after inoculation. Samples (approximately 0.5 g fresh weight of tissue) were ground in a pre-cooled mortar to a fine powder using liquid nitrogen and then homogenized in 1.5 mL 100% methanol. The extracts were sonicated for 10 min and centrifuged in 2 mL Eppendorf tubes for 15 min at 10000 *g* to remove cellular debris. The supernatant corresponding to each sample was divided in two equal portions and dried at 40°C with a flow of nitrogen. One half of the dried residue was resuspended in 900 μL of 50 mM sodium acetate (pH 4.5) and 100 μL of water containing 10 U of almond β-glycosidase (EC 3.2.1.21) (14.3 U/mg, Fluka, Buchs, Zwitzerland) in order to analyse total SA and GA. The other half was resuspended in 900 μL of 50 mM sodium acetate (pH 4.5) and 100 μL of water to analyse free SA and GA. The reactions were incubated overnight at 37°C and then stopped by adding 75 μL of 70% perchloric acid to the incubation mixtures (5% (v/v) final concentration) and maintained at 4°C for 1 h. After centrifugation at 14000 *g* for 15 min to remove polymers, the supernatants were extracted with 2.5 ml of cyclopentane/ethyl acetate (1:1, v/v). The organic upper phase was collected and dried at 40°C under a flow of nitrogen. The residue was resuspended in 200 μL of methanol and filtered through 13-mm nylon 0.45 μm Minispike filters (Waters) prior to HPLC analysis. Twenty μL from the final 200 μL methanolic extract were injected with a Waters 717 autosampler into an analytical reverse-phase Sun Fire C18 5 μm pore size (4.6 mm x 150 mm, Waters) column maintained at room temperature, and equilibrated with 1% acetic acid in water. A 20 min linear gradient of 1% (v/v) acetic acid to 100% methanol at a flow rate of 1 mL/min was applied with a 1525 Waters Binary HPLC pump. The column was washed with 100% methanol during 10 min, and then equilibrated again with 1% acetic acid in water for 10 min. SA and GA were detected with a 2475 Waters Multi λ Fluorescence Detector (λ excitation 313 nm; λ emission 405 nm). Identification and recovery rates of SA and GA were determined by spiking control samples with known amounts of authentic standards, and SA and GA were quantified using the Waters Empower software by constructing a standard curve with authentic standard compounds. Final data were obtained by correcting for losses in the extraction procedure, and recovery of metabolites ranged between 50 and 80%.

### RNA extraction and Northern Blot Analysis

Total RNA of tomato leaf tissue was isolated using TRIzol reagent (Invitrogen) according to the manufacturer’s protocol. To detect the presence of CEVd, 40 μg of total RNA were separated in a 5% polyacrylamide denaturing gel, while 80 μg of total RNA and 17% polyacrylamide denaturing gels were used to detect the VdsRNAs. RNAs were transferred onto Nytran (Schleicher & Schuell) membranes and fixed by UV irradiation. Negative strand specific riboprobes for CEVd were generated by in vitro RNA transcription of viroid cDNA clones in the presence of [α-^32^P] UTP. Prehybridization, hybridization (at 70°C for the CEVd detections and at 35°C for the VdsRNAs analysis) and washes were carried out using the procedure previously described [33]. Equal loading was confirmed by ethidium bromide staining and UV irradiation (S1 Fig).

### ELISA

TSWV detection by ELISA was performed according to Soler et al. [32]. Briefly, the microtiter plates were incubated with a dilution of 1:500 polyclonal antibody of TSWV (BR-01, Loewe) for 4 h at 37°C and washed in 0.15 M phosphate-buffered saline, pH 7.4 (PBS), containing 0.05% Tween 20. Plant extracts were prepared in the conjugate buffer (PBS+ 2% polyvinylpyrrolidone 40000) and incubated in coated plates overnight at 4°C. Alkaline phosphatase-conjugated antibody (1:500 dilution) was incubated 4 h at 37°C and the *p*-nitrophenyl phosphate substrate was incubated for 30 min at room temperature. The absorbance was measured with a Titertek Multiskan MCC/340 photometer (405 nm).

### Extraction procedure and HPLC/ESI-MS analysis of phenolics

Extraction of methanol-soluble HCAA from tomato leaflets was done as described [9, 34]. Appropriate leaf tissues from Money Maker or NahG plants infected with CEVd or TSWV and their corresponding mock-inoculated controls were harvested at the indicated time points after inoculations or treatments, immediately ground to a fine powder in liquid nitrogen, and stored at −80°C until use. An aliquot of 0.5 g of leaf powder mixture from each plant sample was homogenized in 1.5 mL of methanol using a mortar and pestle. The extraction mixture was vortexed for 1 min, and then sonicated for 10 min and centrifuged at 14000 *g* for 15 min to remove cellular debris. The pellet was resuspended in 1 mL of methanol, and the same steps were repeated as above. Both supernatants (total volume 2.5 mL) were transferred to 5-mL glass tubes and dried under a flow of nitrogen at 40°C. The residue was dissolved in 500 μL of methanol and filtered through 13 mm Nylon 0.45 μm Minispike filters (Waters). The solvent was evaporated and the residue dissolved again in 100 μL of methanol. All steps of the extraction were performed in the dark to avoid cis/trans light-induced isomerization of phenylpropanoid double bonds. A 20 μL aliquot from the final 100 μL sample was injected into an analytical reverse-phase Sun Fire 5 μm C18 column (4.6 mm ×150 mm, Waters) equilibrated in 1% acetic acid at 25°C, as previously described [34]. Eluents were 1% acetic acid (eluent A) and methanol (eluent B). A linear gradient starting with 100% eluent A and 0% eluent B was applied over 20 min at a flow rate of 1 ml min^−1^ with a Waters 1525 HPLC binary pump connected to a Waters 2996 photodiode array detector (PDA) and a Waters ZMD mass spectrometer equipped with an electrospray ionization (ESI) source. After washing the column with 100% methanol for 5 min, the initial conditions were again applied and the column was allowed to equilibrate with 1% acetic acid for 10 min. A post-PDA split delivered approximately 25% of the flow to the Waters ZMD mass spectrometer. The source parameters of the mass spectrometer for ESI in negative and positive mode were the following: capillary voltage 2500 V, cone voltage 20 V (negative mode) or 30 V (positive mode), extractor 5 V (negative mode) or 7 V (positive mode), RF Lens 0.5 V, source block temperature 100°C and desolvation gas temperature 300°C. The desolvation and cone gas used was nitrogen at a flow rate of 300 L/h and 50 L/h, respectively. Other mass spectrometer conditions were: low mass resolution 13.5 (negative mode) or 16.6 (positive mode), high mass resolution 13.5 (negative mode) or 16.6 (positive mode), ion energy 0.5 (negative mode) or 0.2 (positive mode), and multiplier 650. A full scan range from mass-to-charge ratio [m/z] 100–800 at 1 s per scan was used for ESI data acquisition. Compounds were quantified from the data recorded and analyzed with the Masslynx Waters software by constructing standard curves with authentic synthesized standards using ESI in positive mode.

### Synthesis of caffeoylputrescine and feruloylputrescine

Chemicals were obtained from commercial suppliers and used without further purification. Caffeic acid, ferulic acid, putrescine (hydrochloride form), and N, N-dicyclohexylcarbodiimide (DCC) were obtained from Sigma–Aldrich and tetrahydrofurane (THF) was from J. T. Baker. The identified trans-HCAA were synthesized by condensation of caffeic and ferulic acids with putrescine in the presence of DCC as the dehydrating agent, following the method described [9]. A solution of DCC (0.5 mmol) in 5 mL of THF was added to a mixture of caffeic acid or ferulic acid (0.5 mmol each) dissolved in 10 mL of THF), and 1 mmol of putrescine dissolved in 10 mL of H_2_O containing 1 mL of pyridine. Then, the reaction mixture was stirred overnight at room temperature. After removal of the solvent, the reaction mixture was diluted with 10 mL of 1% acetic acid in H_2_O and extracted with ethyl acetate. The aqueous phase was neutralized with 1 M NaOH and evaporated to dryness to yield a yellow viscous oil (50 mg). Under the same experimental conditions used for the analysis of phenolics, retention times of synthesized HCAA were the following: 5.05 min for caffeoylputrescine and 6.28 for feruloylputrescine. The mass spectra corresponding to CaP and FP from total ion current chromatograms showed protonated (M+H)^+^ fragment ions of a mass-to-charge ratio *m/z* equal 251 and 265, respectively. The product ion scan spectrum of CaP and FP gave ions at *m/z* 163 and 177, which are daughter ions, characteristic of the caffeic and ferulic moieties, respectively. The maximum wavelength observed for both amides were 293 and 317 nm. The synthesized compounds were found to be identical to the unknown amides present in tomato leaves upon pathogen infection, as judged by comparison of their retention times in the analytical HPLC chromatograms, as well as UV and MS spectra.

### Protein extraction and electrophoresis analysis

Crude protein extracts were prepared from tomato leaf tissues corresponding to CEVd- and TSWV-infected, and mock-inoculated plants at different times after pathogen inoculation as follows: leaf tissue was homogenised in 50 mM Tris-HCl, pH 7.5, containing 15 mM 2-mercaptoethanol (3 mL buffer per gram of fresh weight). The homogenate was transferred to 1.5 mL Eppendorf tubes and centrifuged at 12000 *g* for 15 min at 4°C. Twenty μL samples of the clarified supernatant were then analysed by SDS-PAGE using 14% polyacrylamide gels as described [6]. Coomassie Brilliant Blue R-250 (Sigma) in 10% acetic acid and 10% 2-propanol was used for gel staining. For immunoblot analyses, proteins from duplicate gels were transferred to OPTITRAN BAS-85 nitrocellulose membranes (Schleicher and Schuell) using a Hoefer Semiphor electrotransfer unit (Pharmacia Biotech). Membranes were blocked overnight with 2% non-fat milk in Tris-buffered saline and then incubated with rabbit antisera raised against tomato PR1 [35] or P23 [36]. Incubations took place at room temperature for 1 h using a 1:1000 dilution of anti-PR1 antiserum or a 1:500 dilution of anti-P23 antiserum. The secondary antibody used was alkaline phosphatase-conjugated goat anti-rabbit (Promega), and antigen-antibody complexes were visualized using Nitro Blue Tetrazolium and 5-bromo-4-chloro-3-indolyl phosphate (Sigma) as substrates for alkaline phosphatase.

### Ethylene measurements

Money Maker and NahG leaflets showing symptoms of CEVd or TSWV infection, and the corresponding controls were harvested at the indicated time points after infection, their fresh weight was determined (0.4–0.5 g on average), and they were enclosed in gas-tight 10-ml glass vials fitted with a septum. The vials were placed back in the growth chamber for 5 hours and 400 μL of the gas phase in the vial was analyzed by gas chromatography on a 4890A Hewlett Packard gas chromatograph fitted with a flame ionization detector (FID) with a Teknokroma capillary column (2 m x 1/6” OD x 1 mm ID, Alumina F1 80/100). The carrier gas was helium with a pre-column pressure of 140 kPa. The temperatures of the injector and detector were set at 200°C, and the temperature of the oven was set at 80°C. The retention time of the ethylene peak under these conditions was 2.5 minutes. For each time point, three replicates were analyzed and the amount of ethylene was calculated from the data recorded and analyzed with the Masslynx Waters software by constructing a standard curve with authentic ethylene.

### Statistical analyses

The symptomatology of every plant, which was monitored at the indicated time points and scored according to symptom severity, was statistically analyzed using a Kruskal-Wallis test (non-parametric test equivalent to the one-way ANOVA). This test was used to compare NahG and Moneymaker symptomatologies at every time point, for both infections. Different letters indicate significant differences (*p* < 0.05) between the Money Maker and NahG infected plants.

The “Infectivity Index” consists of the total number of days that each plant presents symptoms, thus providing a measure of the delay in the onset of symptoms [37]. Data from a representative experiment from three independent assays were used to perform statistical analysis by using the Mann-Whitney non-parametric test. This test was used to compare infectivity indexes of Moneymaker and NahG plants, as well as NahG and BTH-treated NahG plants, for both infections. A *p* value < 0.05 was considered as statistically significant.

The IBM SPSS v.19 package was used for all the statistical analysis.

## Results

### Accumulation of SA and GA upon CEVd or TSWV infection in Money Maker and NahG tomato plants

**Fig 1:**
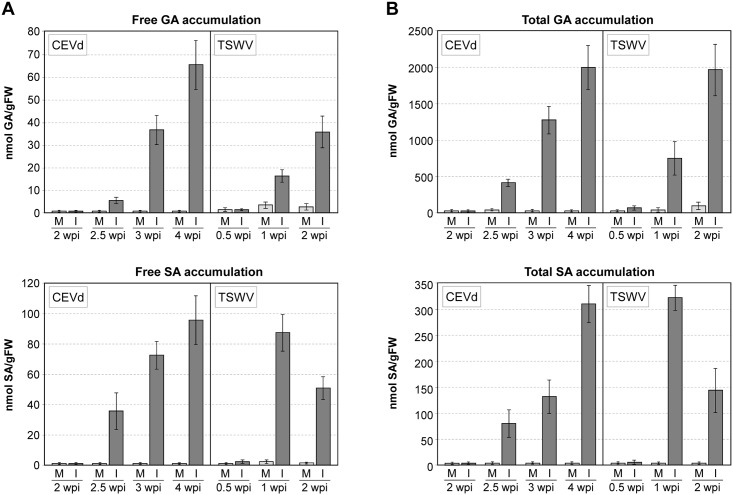
Accumulation of phenolic compounds in Money Maker plants infected with citrus exocortis viroid (CEVd) or tomato spotted wilt virus (TSWV). Levels of free **A)** and total **B)** gentisic acid (GA) and salicylic acid (SA) in mock-inoculated and infected Money Maker tomato leaves. Plants were inoculated with either CEVd or TSWV and samples were collected at the indicated times (weeks post-inoculation, wpi) for both infected (I) and mock-inoculated (M) leaves, and then analyzed by HPLC-fluorescence detection. Total SA is the sum of free SA and SA glucoside and total GA is the sum of free GA and GA xyloside. Data correspond to the mean ± SD of three biological replicates of one representative experiment. The experiments were repeated at least 3 times.

Infection of Money Maker tomato plants with either CEVd or TSWV induced a strong accumulation of both free and total SA and GA, which started at very low basal levels and increased notably upon appearance of the first mild symptoms of the disease. Fig 1 depicts a time-course study showing the dramatic accumulation of free and total SA and GA in CEVd- and TSWV-infected Money Maker tomato leaves during the disease progression. The levels of GA increased throughout both infection processes. The accumulation of SA was qualitatively similar to the GA accumulation in the CEVd infection, but a maximum level of SA was reached at 1 week after the TSWV inoculation. Comparing free and total levels of these phenolics, we observed that about 30% of SA and more than 95% of GA accumulated in infected leaves was in the conjugated form.

**Fig 2:**
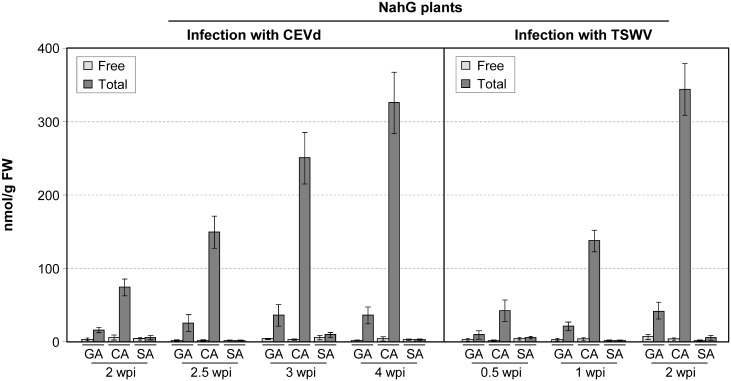
Accumulation of phenolic compounds in NahG plants infected with citrus exocortis viroid (CEVd) or tomato spotted wilt virus (TSWV). Levels of free and total gentisic acid (GA), salicylic acid (SA) and catechol (CA) in infected NahG tomato leaves. Plants were inoculated with either CEVd or TSWV and samples were collected at the indicated times (weeks post-inoculation, wpi), and then analyzed by HPLC-fluorescence detection. Total phenolics are the sum of free and glycosylated forms. Data correspond to the mean ± SD of three biological replicates of one representative experiment. The experiments were repeated at least 3 times.

Fig 2 shows that both CEVd- or TSWV-infected NahG tomato plants did not accumulate SA and only small amounts of GA could be measured during the infections. A pronounced time-dependent linear synthesis of conjugated catechol, which is the SA degradation product in NahG plants, was observed upon both infections, reaching levels similar to those of total SA in Money Maker plants (Fig 1B).

### NahG tomato plants are hypersusceptible to CEVd and TSWV pathogens

**Fig 3:**
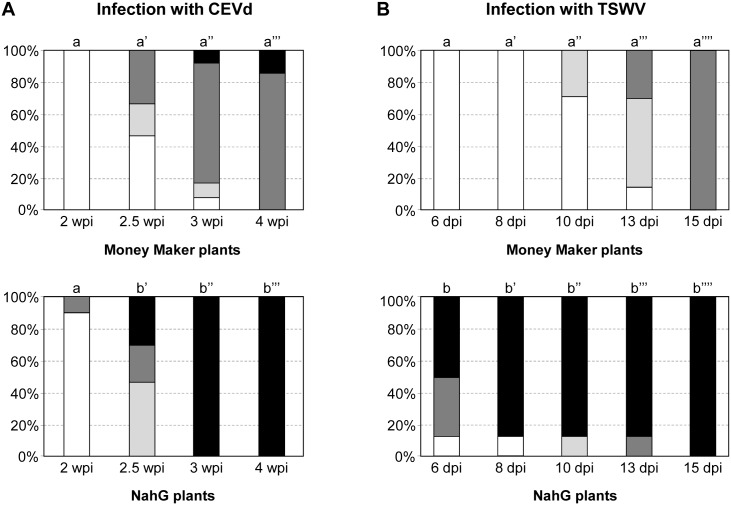
Disease severity of infected Money Maker and NahG tomato plants. Symptoms scale provoked by **A)** Citrus Exocortis Viroid (CEVd) and **B)** Tomato Spotted Wilt Virus (TSWV). Symptomatology was scored at 2, 2.5, 3, and 4 weeks post-inoculation (wpi) with CEVd and at 6, 8, 10, 13, and 15 days post-inoculation (dpi) with TSWV using the following scale: symptomless (white), moderate (grey), severe (dark gray), and very severe (black). Data of a representative experiment are shown. A Kruskal-Wallis analysis was performed and different letters indicate significant differences (*p* < 0.05) between the Money Maker and NahG plants. The experiments were repeated at least 3 times.

**Fig 4:**
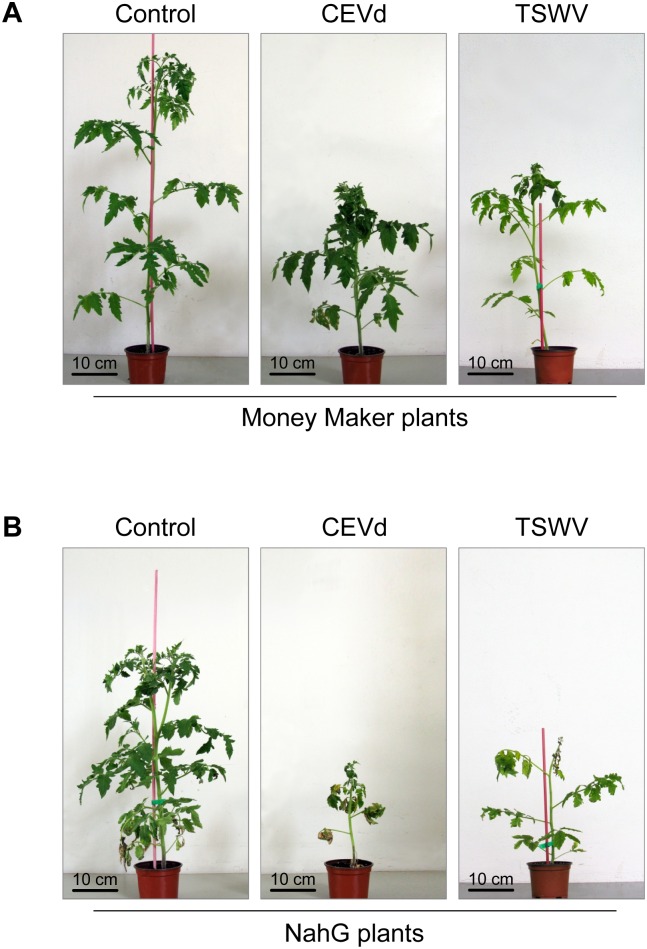
Growth of representative Money Maker and NahG tomato plants following inoculation with citrus exocortis viroid (CEVd) and tomato spotted wilt virus (TSWV). **A)** Mock-inoculated Money Maker plant (Control), Money Maker plant 4 weeks after CEVd-inoculation (CEVd), and Money Maker plant 15 days after TSWV-inoculation (TSWV). **B)** Mock-inoculated NahG plant (Control), NahG plant 4 weeks after CEVd-inoculation (CEVd), and NahG plant 15 day after TSWV-inoculation (TSWV).

Control and CEVd-infected Money Maker and NahG plants were visually inspected for symptom development at the indicated time points. The onset of symptoms occurred on the tip tissues (apex and very new young leaves) of CEVd-infected plants. Symptoms were scored from mild (rugosity, reduced size and downward curling of the tip leaves) to very severe symptoms (bunchiness and epinasty of the leaves, marked growth retardation and stunting of the plant caused by shortening of internodal length). Two weeks after CEVd-inoculation, severe symptoms were already evident in 10% of the NahG plants, while all Money Maker plants were totally free of symptoms (Fig 3A). The differences were even more pronounced at 3 weeks post-inoculation. At that time, all NahG plants exhibited very severe symptoms, while similar symptoms were observed only in the 10% of Money Maker plants. Fig 4A and 4B depict representative CEVd-infected Money Maker and NahG plants, respectively, at 4 weeks after inoculation.

Symptoms in tomato plants infected with TSWV consisted of leaf reduction and distortions (upward and downward leaf curling of the leaf margins), and as the disease progresses, a pronounced threadlike foliage, epinasty of the leaves and stunting of the whole plant was observed (Fig 4A and 4B). The first symptoms in TSWV-infected NahG plants were evident very early. In fact, at 6 days after inoculation, while 100% of Money Maker plants were symptomless, 90% of NahG-infected plants displayed symptoms, with 50% of them showing very severe symptoms (Fig 3B). One week later, while nearly 90% of the TSWV-infected NahG plants showed very severe symptoms, less than 30% of the TSWV-infected Money Maker plants presented only moderate symptoms of the disease (Fig 3B). It is important to note that during all stages of the infection, TSWV-infected NahG plants displayed much stronger symptoms than those of TSWV-infected Money Maker tomato plants.

The results shown in Fig 3 were analyzed statistically (see Materials and Methods). At every time point and for both infections, significant differences between Money Maker and NahG plants were observed, with the exception of CEVd-infected plants at 2 weeks post-infection, when the symptoms still were not evident (S1 and S2 Tables).

### BTH reverses the enhanced susceptibility of NahG plants to CEVd or TSWV

To further investigate whether the enhanced susceptibility of NahG tomato plants to CEVd or TSWV is due to the lack of SA accumulation, we studied the ability of BTH to restore the basal resistance of tomato to these pathogens. Based on previously reported BTH concentrations for foliar treatments in different plants including tomato [38–40], 1 mM BTH treatments were employed. This concentration had no visible effect on plant development in Money Maker plants and prevented the necrotic phenotype of NahG plants, which were grown at high light conditions for correct viroid replication [14].

**Fig 5:**
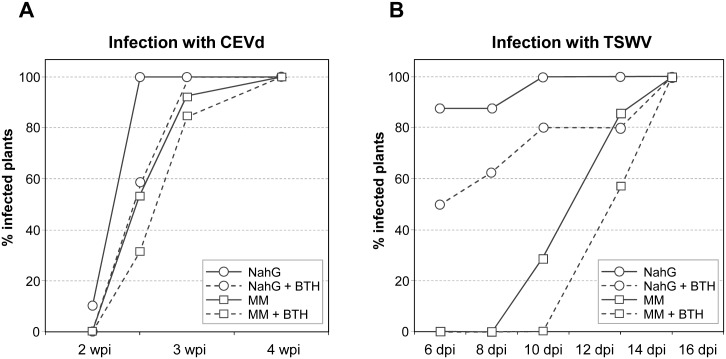
Disease development in Money Maker and NahG plants infected with A) CEVd or B) TSWV. Plants were treated either with water (continuous line) or BTH (discontinuous line). Evolution of the number of tomato plants showing symptoms at the indicated days post-inoculation (dpi). Data correspond to one representative experiment.

**Fig 6:**
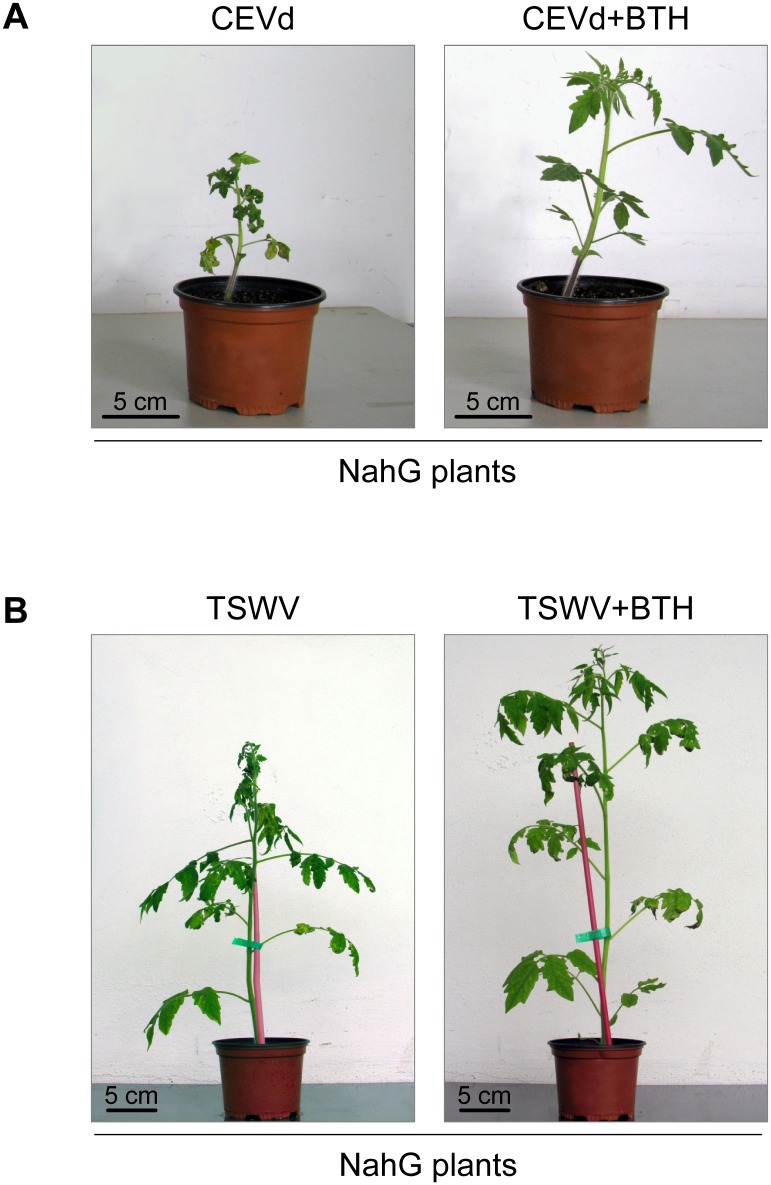
Growth of NahG plants following treatment with BTH and CEVd or TSWV inoculation. **A)** Representative phenotype observed in infected plants 4 weeks after CEVd inoculation (CEVd), and in equivalent plants that had been pre-treated with 1 mM of BTH (CEVd+BTH). **B)** Representative phenotype observed in TSWV-infected plants 15 days after inoculation (TSWV), and in equivalent plants that had been pre-treated with 1 mM of BTH (TSWV+BTH).

Application of BTH in CEVd-inoculated NahG plants markedly delayed the initial appearance of symptoms (Fig 5A). At 2 weeks post-inoculation, 10% of the CEVd-infected NahG tomato plants presented symptoms, while CEVd-infected NahG plants pre-treated with BTH remained totally asymptomatic. More strikingly, at 2.5 weeks after inoculation, all NahG plants showed symptoms, while BTH treatments reduced the percentage of NahG plants showing symptoms to less than 60%. These results indicate that BTH is able to rescue, at least partially, the hypersusceptibility phenotype of NahG plants. Fig 6A illustrates a representative BTH-restored phenotype of CEVd- infected NahG plants. At 3 weeks post-inoculation, the BTH-mediated protection effect is no longer observed and symptoms appeared in all viroid-infected NahG plants.

Qualitatively comparable results were obtained in TSWV-infected NahG tomato plants, but with a much more pronounced effect during the early time points, probably because of the rapid progression of the infection process caused by the virus. As observed in Fig 5B, about 90% of TSWV-infected NahG plants showed disease symptoms 6 days after inoculation, while only about 50% of the TSWV-infected NahG plants previously treated with BTH showed symptoms at this time. Fig 6B shows this reduction in the severity of the symptoms in TSWV-infected NahG plants pre-treated with BTH. The protective effect mediated by BTH was still relevant at 10 and 13 days post-inoculation, while no effect was observed at day 15 after inoculation. Additionally, the BTH-conferred resistance observed in the hypersusceptible NahG plants was also observed in Money Maker plants against both infections, but to a lesser extent (Fig 5).

To verify the statistical significance of the differences shown in Fig 5, the “Infectivity Index” was used as a measure of the delay in the onset of symptoms. Infectivity indexes from Money Maker and NahG plants infected either with CEVd or TSWV and treated with BTH were used. We observed statistically significant differences among the infectivity indexes for Money Maker and NahG plants infected with CEVd or with TSWV. Moreover, the effect of BTH on NahG plants was also statistically significant (S3 Table).

**Fig 7:**
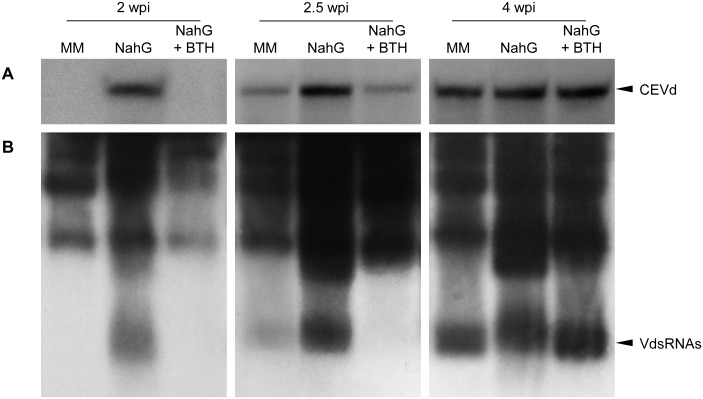
Detection of CEVd levels in infected Money Maker, NahG and BTH-treated NahG plants. Panels A and B show the Northern blot analyses to detect Citrus Exocortis Viroid (CEVd) and CEVd-specific small RNAs (VdsRNAs), respectively, at 2, 2.5 and 4 weeks after inoculation (wpi). RNAs were separated in a 5% or 17% denaturing polyacrylamide gel, then transferred and hybridized with radioactive negative strand CEVd riboprobes. CEVd and its corresponding small RNAs are denoted with an arrow.

To study the possible relationship between the observed symptomatology and the titre of the pathogens, the levels of CEVd and TSWV were measured in infected Money Maker plants, as well as in both hypersusceptible and BTH-treated infected NahG plants. To this end, total RNA extracts at different stages of the infection were used to analyse the CEVd content and the presence of small viroid-derived RNA, by Northern Blot. The results depicted in Fig 7A demonstrate that in infected NahG plants, CEVd rapidly accumulated at week 2 after inoculation, coinciding with the onset of symptoms, while no detectable CEVd appeared in infected Money Maker or BTH-treated NahG leaves, both not yet bearing symptoms. At 2.5 weeks post-inoculation, when CEVd-infected Money Maker and BTH-NahG plants began to show the first mild symptoms, CEVd accumulation was detected, although at lower levels than those observed in untreated NahG plants. At 4 weeks after inoculation, CEVd in Money Maker and BTH-NahG plants reached similar levels as those observed in NahG plants. Regarding the small viroid RNA accumulation, a pattern similar to that described for CEVd was observed (Fig 7B).

**Fig 8:**
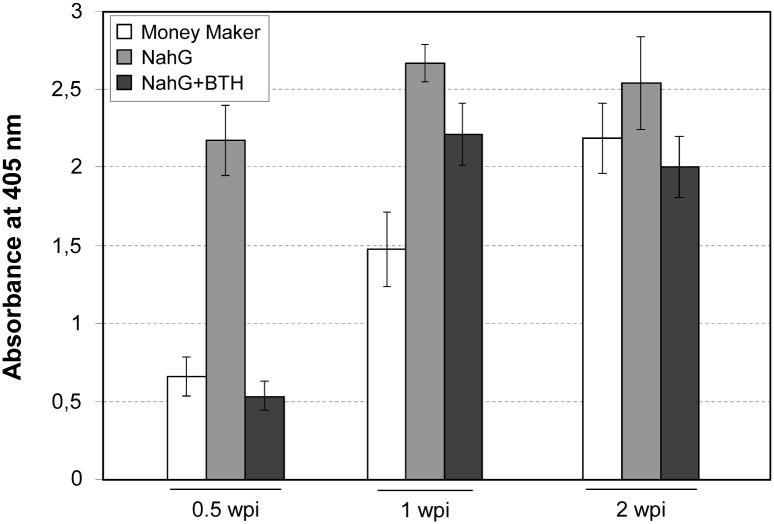
Detection of TSWV levels in infected Money Maker, NahG and BTH-treated NahG plants. Quantitative ELISA to detect TSWV at 0.5, 1 and 2 wpi. A polyclonal antibody against TSWV (BR-01, Loewe) and an alkaline phosphatase conjugated antibody were used as primary and secondary antibodies, respectively. Immunodetection was carried out by using p-nitrophenyl phosphate as a substrate and the absorbance was measured at 405 nm. Data correspond to the mean ± SD of three biological replicates of one representative experiment.

The analysis of the TSWV content showed similar results (Fig 8). The development of symptoms in infected plants correlated with the content of the virus at the beginning of the infection process (0.5 weeks post-inoculation), being the TSWV levels lower in both Money Maker and BTH-treated NahG plants, when compared with the non-treated NahG plants. However, at 2 weeks post-inoculation, the amount of virus was similar in all the plants, while a different degree of symptom development was observed (Fig 3B).

Therefore, infected NahG plants presented an earlier accumulation of both CEVd and TSWV, when compared with Moneymaker or BTH-NahG plants, displaying a correlation between the levels of pathogen accumulation and the symptomatology. These differences appeared to vanish as the infection progressed.

### Phenylpropanoids in Money Maker and NahG tomato plants

We have previously reported that the Rutgers tomato cultivar accumulated various hydroxycinnamic acid amides (HCAA) upon infection with the bacterial pathogen *Pseudomonas syringae* pv. *tomato* [8, 9]. Here we wanted to explore whether some of these metabolites also accumulated in Money Maker and NahG plants after inoculation with CEVd or TSWV. A simple comparison between the HPLC-chromatograms corresponding to methanolic extracts from mock-inoculated and those of CEVd- or TSWV-infected Money Maker or NahG tomato leaves showed four prominent peaks. Two of these peaks corresponded to *de novo* synthesis of the neutral HCAA *p*-coumaroyltyramine (CT) and feruloyltyramine (FT). The other two peaks were identified as the basic water-soluble HCAA polyamine conjugates caffeoylputrescine (CaP) and feruloylputrescine (FP). All the compounds were unambiguously identified by comparison of their electrospray ionization mass spectra with those of previously synthesized CT and FT [8], and those synthesized in this work, CaP and FP (see Materials and Methods). In all cases, putative HCAA had identical retention time, UV and mass spectra to that of authentic synthesized HCAA.

**Fig 9:**
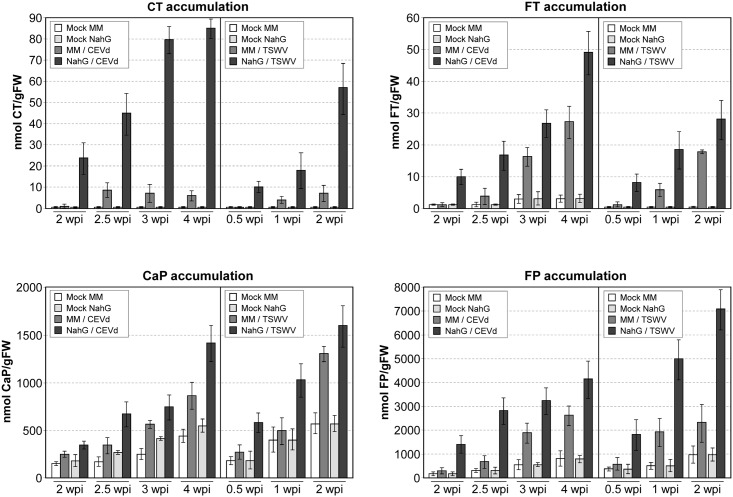
Effect of Citrus Exocortis Viroid (CEVd) and Tomato Spotted Wilt Virus (TSWV) infections on levels of p-coumaroyltyramine (CT), feruloyltyramine (FT), caffeoylputrescine (CaP), and feruloylputrescine (FP) in Money Maker and NahG tomato leaves. Plants were inoculated with either CEVd or TSWV and samples were collected at the indicated times (weeks post-inoculation, wpi) for both infected and mock-inoculated leaves, and then analyzed by HPLC/ESI-MS. Data correspond to the mean ± SD of three biological replicates of one representative experiment. The experiments were repeated at least 3 times.

Fig 9 shows the time course of the induction of the amides of tyramine (CT, FT) and putrescine (CaP and FP) in Money Maker and NahG tomato leaves upon infection with CEVd or TSWV. No detectable basal amounts of CT and FT were found in mock-inoculated plants, even in 5–6 week old plants. In contrast, significant basal levels of CaP and FP were found in tomato leaves. The initial detection of all the amides coincided with the appearance of the first symptoms, and increased in a time-dependent manner in both Money Maker and NahG plants infected with CEVd or TSWV. Although qualitatively quite similar, the magnitudes of the amide accumulation in leaf tissues presented notable quantitative differences, depending on the specific plant-pathogen interaction. In both infections, the maximum level of amides was reached in NahG plants, correlating with the hypersusceptibility and symptom severity observed in these plants, as compared with those exhibited by Money Maker plants.

### Basic PR1 and P23 are highly induced in infected NahG tomato plants

**Fig 10:**
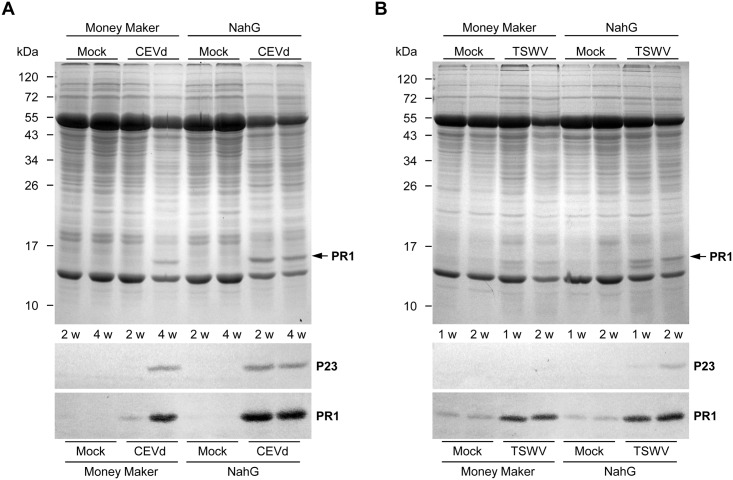
Accumulation of Pathogenesis-Related (PR) proteins in Money Maker and NahG tomato plants upon viroid and virus infection. Plants were inoculated with either Citrus Exocortis Viroid (CEVd, **A)** or Tomato Spotted Wilt Virus (TSWV, **B)**, and samples were collected at the indicated times (weeks post-inoculation, wpi) for both infected and mock-inoculated plants. Crude extracts were separated by SDS-PAGE and the presence of PR1 and P23 was detected by immunoblot. Upper panels correspond to Coomassie Blue stained SDS-PAGE gels (14% acrylamide). Lower panels show the immunostaining of P23 (23 kDa) and PR1 (14 kDa).

To elucidate whether CEVd- or TSWV-infected NahG tomato plants, which are unable to accumulate SA or GA, are also compromised in the induction of defence proteins, accumulation of PR1 and P23 was studied by western blot at two time points after inoculation. PR1 and P23 are, respectively, 14 kDa and 23 kDa proteins which are rapidly induced in Rutgers tomato plants when they are infected with CEVd [41]. As shown in Fig 10A, a strong induction of PR1 isoforms was inmuno-detected at 2 weeks post-inoculation in CEVd-infected NahG tomato plants, while only a slight accumulation of this defence protein was observed in the corresponding Money Maker samples. Four weeks after inoculation, a considerable accumulation of PR1 isoforms was also detected in Money Maker plants, reaching similar levels to those detected in NahG plants. Therefore, the PR1 accumulation correlates with the appearance of symptoms of viroid disease. Since the acidic PR1 isoform has been described to be SA-dependent in tomato plants [42], the inmuno-detected PR1 in infected NahG tomato plants probably corresponds to the basic ethylene-responsive isoform [43].

Fig 10B shows that a marked and similar induction of basic PR1 occurred both in TSWV-infected Money Maker and NahG-infected tomato plants, with a slightly higher induction of PR1 in NahG at a later stage of infection, as compared to Money Maker plants. The background observed in mock-inoculated plants could be due to the age of the plants (see Materials and Methods), in agreement with previous results showing enhanced expression of PR in senescent tomato leaves [41].

A qualitatively similar pattern although with lower intensity, was observed for the induction of P23 in both Money Maker and NahG tomato plants infected with CEVd (Fig 10A). Regarding TSWV infection, P23 accumulation was only detected in NahG plants (Fig 10B).

### Ethylene production is dramatically increased in infected NahG plants

**Fig 11:**
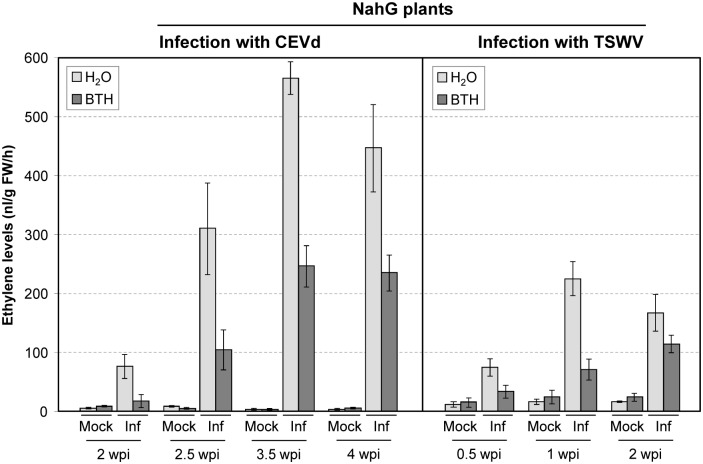
Time course of ethylene production in NahG tomato leaves infected with Citrus Exocortis Viroid (CEVd) or Tomato Spotted Wilt Virus (TSWV) and their corresponding controls. Samples were collected at the indicated times (weeks post-inoculation, wpi) for both mock-inoculated (Mock) and infected (Inf) plants, and ethylene was measured with a gas chromatograph fitted with a flame ionization detector (FID). Data correspond to the mean ± SD of three biological replicates of one representative experiment. The experiments were repeated at least 3 times.

Convincing evidence has been presented showing that ethylene is also involved in the response of Rutgers tomato to CEVd infection [44]. These studies and the results presented here on the induction of basic PR1 in NahG plants infected with both CEVd or TSWV, prompted us to test whether SA-enhanced resistance to CEVd or TSWV was correlated with ethylene signalling. In order to accomplish this goal, we measured ethylene production during the progression of the disease in viroid- and virus-infected NahG tomato plants. Fig 11 shows the evolution of ethylene biosynthesis in NahG plants upon CEVd and TSWV infections during 4 and 2 weeks after inoculation, respectively. In striking contrast with infected Money Maker plants, a dramatic increment in ethylene synthesis occurred in NahG plants inoculated with either pathogen. In CEVd-infected NahG tomato plants ethylene is emitted coinciding with the onset of symptom development, reaching a maximum at 3.5 weeks after inoculation. A qualitatively similar pattern of ethylene induction was found in TSWV-infected NahG plants, with a maximum accumulation at 1 week after inoculation.

Moreover, Fig 11 also shows that the increased ethylene evolution in both CEVd- and TSWV-infected NahG plants treated with BTH was lower than that observed in infected plants that had not been pre-treated, correlating with the delay and reduction of disease development in the BTH-treated plants.

## Discussion

In this work, we have studied the role of SA in the basal resistance of tomato plants. We have observed that NahG tomato plants, unable to accumulate SA, are hypersusceptible to CEVd and TSWV, presenting an earlier accumulation of the pathogens, which is accompanied by an early and more intense accumulation of hydroxycinnamic amides (*p*-coumaroyltyramine, feruloyltyramine, caffeoylputrescine, and feruloylputrescine), ethylene and the defence-related proteins basic PR1 and P23. In addition, BTH treatments completely or partially rescue the hypersusceptibility phenotype of NahG plants. Our results clearly indicate that SA is an important component of basal resistance of tomato plants.

Infection of Money Maker tomato plants with either CEVd or TSWV induced a strong accumulation of SA and GA, as previously described in other compatible interactions [7, 34]. Moreover, GA accumulation occurred later than that of SA, all of this in agreement with the fact that SA is the metabolic precursor of GA in tomato plants [7]. On the other hand, the high levels of SA and GA in their conjugated form confirmed that both phenolics are rapidly metabolized to their β-glycosides [45, 46].

The hypersusceptibility of NahG plants has been described in many other plant-pathogen interactions [4, 15, 16, 42]. However, this is the first time that these transgenic plants have been used to demonstrate the essential role of SA in basal resistance against viroids and TSWV. Our results differ from those reported regarding the response of NahG tomatoes against the bacterial pathogen *Xanthomonas campestris* [22]. In this compatible interaction, NahG plants displayed a reduction in disease symptoms, thus indicating that the basal resistance of tomato plants to this bacterial pathogen is SA-independent.

Several lines of evidence have revealed a possible overlap between RNA-silencing pathways and SA-mediated defence [5, 47]. In this respect, exogenous treatments with SA have been described to induce RNA silencing-related genes in tomato [37]. The accumulation of VdsRNAs derived from CEVd in NahG plants appear to indicate that SA is not required for the activation of RNA-silencing mechanisms in tomato. Further studies to compare the activation of RNA silencing-related genes in Money Maker and NahG plants infected by CEVd could be performed in order to better understand the role of SA in the activation of these silencing mechanisms.

Regarding the accumulation of the pathogen in infected NahG plants, we have observed that the earlier appearance of symptoms correlates with the presence of detectable levels of viroid or virus in inoculated plants. However, the greater intensity of symptoms in infected NahG plants does not correlate with a higher concentration of viroid or virus in these plants at later time points. In relation with this, other authors reported results that vary depending on the specific host-pathogen combinations. For example, Potato Virus X accumulates to higher amounts in NahG potato plants than in wild type controls and this positively correlated with the intensity of symptoms [48] and, conversely, symptom severity in viroid-infected plants was, in general, not correlated with the levels of viroid RNAs [49]. The striking differences in the appearance and intensity of symptoms in NahG and Money Maker tomato leaves harbouring equivalent amounts of pathogen at the last time points may suggest additional mechanisms of host involvement in the modulation of symptom development following CEVd or TSWV inoculation.

Interestingly, we have observed that BTH treatments successfully rescue the hypersusceptibility phenotype of NahG plants against CEVd or TSWV by reducing the pathogen titres and the disease incidence and severity at the beginning of the infection. These results are in agreement with those reported by Trejo-Saavedra et al. [40] in pepper crops infected with begomoviruses or those reported in tomato infected with *Cucumber mosaic virus* [50]. Our results strongly suggest that the observed hypersusceptibility is due to the absence of SA, as it can be reverted by BTH. This SA analogue might act as an SA-mediated host defence inducer, rather than as a direct anti-viroid or anti-viral agent, since its antimicrobial activities have been discarded [17, 51]. The duration of the BTH-mediated protective effect against CEVd or TSWV infection decreased over time and impaired viroid and virus replication and symptom development about 10 days after the last treatment. This is in agreement with previous studies performed in tomato plants infected with different pathogens [30, 38, 51–53]. To achieve this protective effect, we have applied BTH before pathogen inoculation, in accordance with Rohilla et al. [54].

We have observed that NahG and Money Maker plants accumulated the hydroxycinnamic amides CT, FT, CaP and FP upon CEVd and TSWV infections. We have also described the accumulation of the tyramine amides in other compatible interactions, such as that produced by *Pseudomonas syringae* in tomato plants [55]. In contrast, large amounts of CT, FT, and FP accumulated only in the incompatible interactions between tobacco and TMV [56], and pepper with *Xanthomonas campestris* [57], being essentially absent in the corresponding compatible interactions. The phenylpropanoid-polyamine conjugate CaP has been described in several plant-insect interactions [58–60]. Here, we show that CaP also accumulates upon virus or viroid infection, expanding the putative defensive role of this amide. The overexpression of key enzymes involved in the HCAAs biosynthesis has been described to confer resistance to different pathogens in tomato, tobacco and rice [55, 61, 62]. Therefore, these hydroxycinnamoyl acid amides could be considered as components of a general defence response of plants in compatible interactions. The observed positive correlation between symptom development and HCAA accumulation suggests that the antimicrobial properties of these compounds are not enough to confer resistance against these RNA pathogens.

Importantly, the fact that HCAAs as well as PRs were found in CEVd- or TSWV-infected NahG plants indicates that the induction of these defensive metabolites and proteins is SA-independent. In accordance with our results, Brading et al. [14] studied PR1 gene expression during *Cf-9*- and *Cf-2*-dependent hypersensitive reactions and showed that NahG plants were able to induce PR1 expression. In addition, NahG potato plants showed increased PR1 expression levels upon challenge with *Potato Virus X* [48]. However, other studies indicate a typical direct correlation between depletion of SA and a clear reduction of the induction of defence genes during the hypersensitive [16, 63, 64] or susceptible responses [22, 42, 65] against different kinds of pathogens.

Some host defence responses to pathogens have been described to be modulated by ethylene [66–68]. In tomato plants, a role for ethylene has been proposed in the regulation of susceptible responses to pathogens [22, 42, 44, 69, 70]. We have analyzed the accumulation of this phytohormone in both CEVd- and TSWV-infected tomato plants. Our results are in agreement with the classically described antagonism between SA and ethylene/jasmonic acid signalling [71], since the absence of SA correlated with a higher accumulation of ethylene in the hypersusceptible NahG plants. In addition, we have observed a positive correlation between ethylene and symptom development, also described in different plant-pathogen interactions [42, 68].

Using NahG tomato plants, here we show the implication of SA as a component of the basal resistance to RNA pathogens and we extend the possible role of ethylene in the susceptible response to them.

## Supporting Information

S1 Fig Ethidium bromide staining of the polyacrylamide gels used for Northern Blot analyses in Fig 7. Arrows indicate the same positions shown in Fig 7A and 7B for CEVd and its corresponding small RNAs, respectively. MM: Money Maker plants. N: NahG plants. N+B: BTH-treated NahG plants.
(TIF)

S1 Table Statistical parameters of Kruskal-Wallis tests for symptomatology indexes of Moneymaker and NahG plants infected with CEVd. (TIF)

S2 Table Statistical parameters of Kruskal-Wallis tests for symptomatology indexes of Moneymaker and NahG plants infected with TSWV. (TIF)

S3 Table Statistical parameters of the Mann-Whitney tests for infectivity of Moneymaker and NahG plants (A) and NahG and BTH-treated NahG plants (B), infected either with CEVd (1) or TSWV (2). (TIF)
