# Supplementary figures and images for: Salicylic Acid Is Involved in the Basal Resistance of Tomato Plants to Citrus Exocortis Viroid and Tomato Spotted Wilt Virus
Source: PLoS One. 2016 Nov 28;11(11):e0166938. doi: 10.1371/journal.pone.0166938 (PMC5125658; doi:10.1371/journal.pone.0166938)

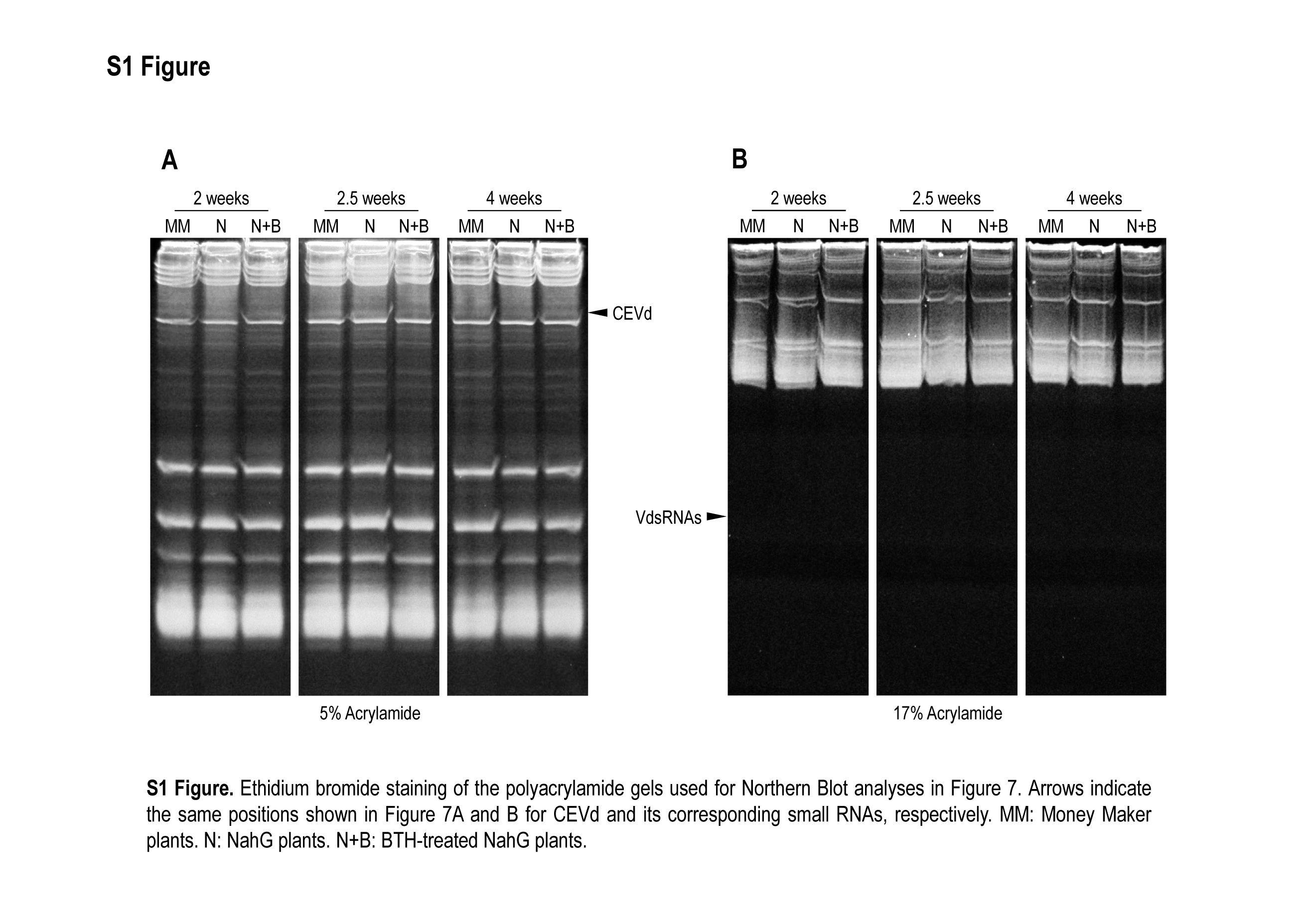

Supplement: S1 Fig — Arrows indicate the same positions shown in Fig 7A and 7B for CEVd and its corresponding small RNAs, respectively. MM: Money Maker plants. N: NahG plants. N+B: BTH-treated NahG plants. (TIF) [file pone.0166938.s001.tif]

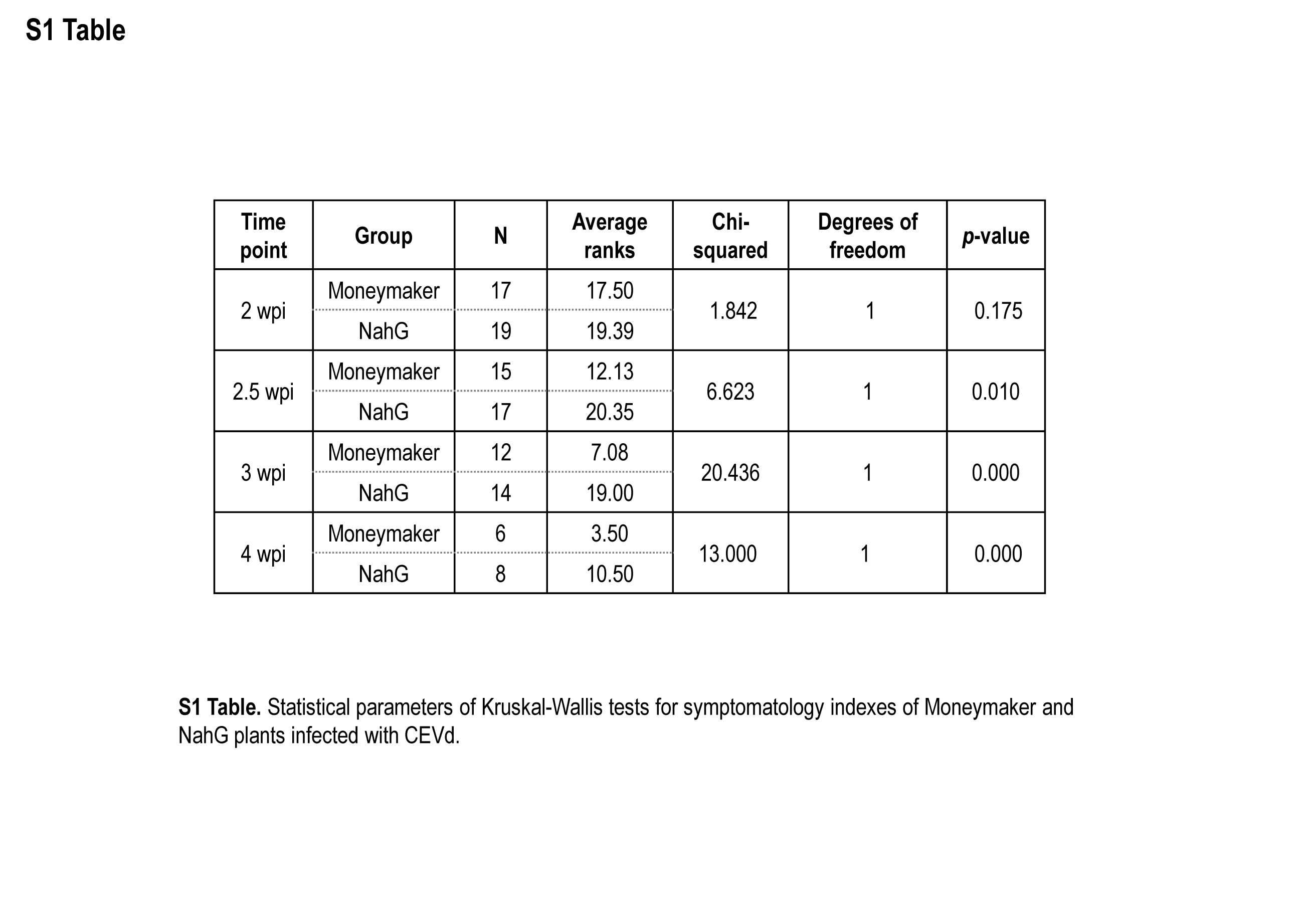

Supplement: S1 Table — (TIF) [file pone.0166938.s002.tif]

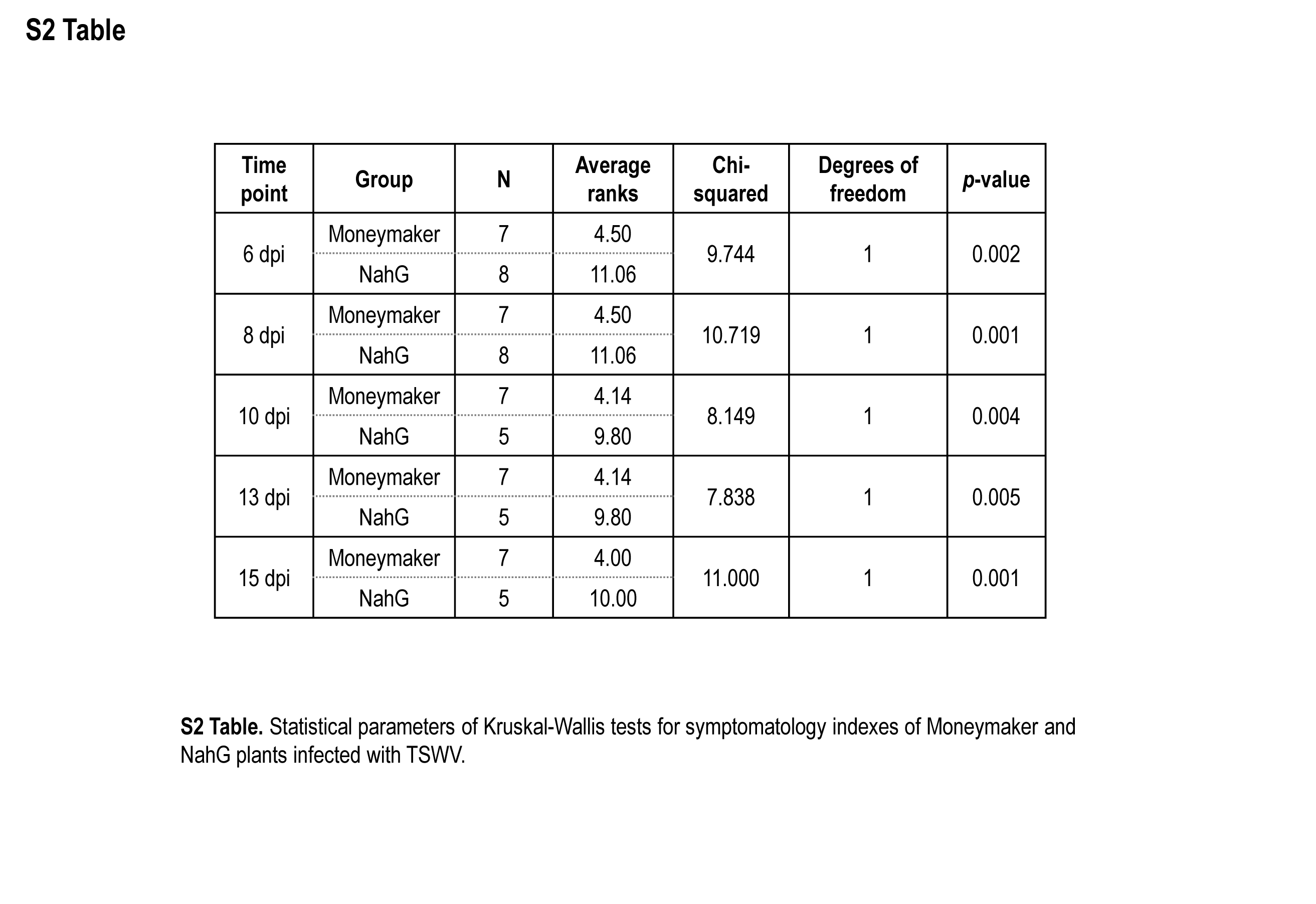

Supplement: S2 Table — (TIF) [file pone.0166938.s003.tif]

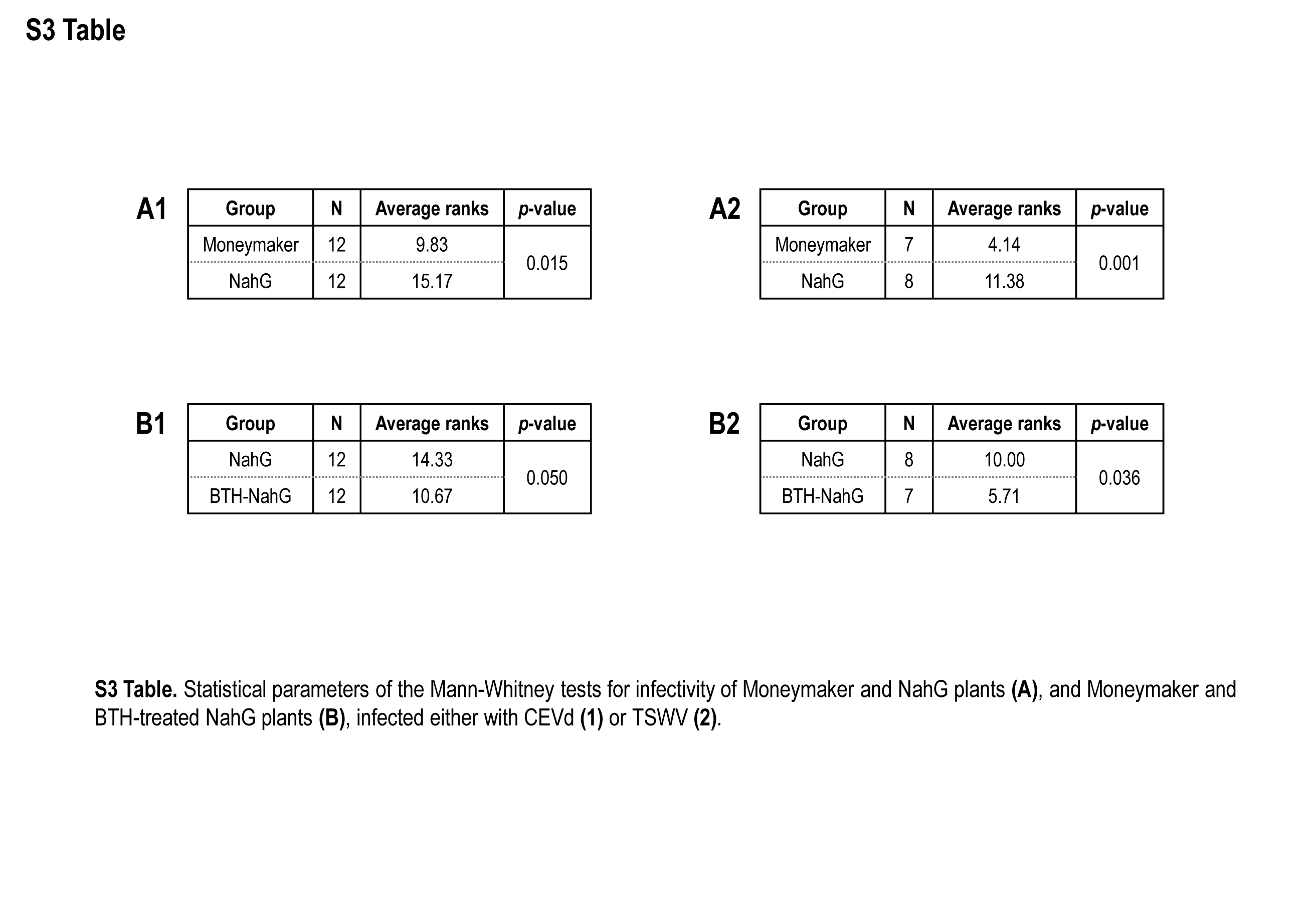

Supplement: S3 Table — (TIF) [file pone.0166938.s004.tif]
